# A Minimal Setup for Spontaneous Smile Quantification Applicable for Valence Detection

**DOI:** 10.3389/fpsyg.2020.566354

**Published:** 2020-12-17

**Authors:** Mauro Nascimben, Thomas Zoëga Ramsøy

**Affiliations:** Neurons Inc, Herlev, Denmark

**Keywords:** facial expressions, valence, surface electromyography, emotion detection, v-commerce metrics

## Abstract

Tracking emotional responses as they unfold has been one of the hallmarks of applied neuroscience and related disciplines, but recent studies suggest that automatic tracking of facial expressions have low validation. In this study, we focused on the direct measurement of facial muscles involved in expressions such as smiling. We used single-channel surface electromyography (sEMG) to evaluate the muscular activity from the Zygomaticus Major face muscle while participants watched music videos. Participants were then tasked with rating each video with regard to their thoughts and responses to each of them, including their judgment of emotional tone (“Valence”), personal preference (“Liking”) and rating of whether the video displayed strength and impression (“Dominance”). Using a minimal recording setup, we employed three ways to characterize muscular activity associated with spontaneous smiles. The total time spent smiling (ZygoNum), the average duration of smiles (ZygoLen), and instances of high valence (ZygoTrace). Our results demonstrate that Valence was the emotional dimension that was most related to the Zygomaticus activity. Here, the ZygoNum had higher discriminatory power than ZygoLen for Valence quantification. An additional investigation using fractal properties of sEMG time series confirmed previous studies of the Facial Action Coding System (FACS) documenting a smoother contraction of facial muscles for enjoyment smiles. Further analysis using ZygoTrace responses over time to the video events discerned “high valence” stimuli with a 76% accuracy. Additional validation of this approach came against previous findings on valence detection using features derived from a single channel EEG setup. We discuss these results in light of both the recent replication problems of facial expression measures, and in relation to the need for methods to reliably assess emotional responses in more challenging conditions, such as Virtual Reality, in which facial expressions are often covered by the equipment used.

## 1. Introduction

Humans display emotional responses in a variety of ways, including changes in facial expressions, skin conductance, heartbeat, brain signals, body temperature, and pulse rate. These measurable body changes are the foundation of Affective Computing, a discipline that studies how to detect emotions and their effect on cognition, perception, learning, communication, and decision-making (Picard, 2003). Among the physiological variables for emotion assessment, those most commonly applied are electroencephalography (i.e., EEG, for a review; Solnais et al., 2013), electrocardiogram (i.e., ECG), galvanic skin response (i.e., GSR, for a review; Caruelle et al., 2019) and heart rate variability (i.e., HRV). However, the most immediate and natural way humans share their emotions are through facial expressions. Seemingly simple, yet highly intricate, the dynamics of facial expressions depend upon a complex architecture of musculature surrounding the calvaria region, orbital opening, mouth, and the nose (Bentsianov and Blitzer, 2004). Among these groups of muscles, the most important one for facial expressions is in the oral area (Cohen, 2006).

Spontaneous activity of facial muscles in response to emotional stimuli is usually referred to as “facial motor resonance” to differentiate it from the movement of facial muscles in imitation of the expression of individuals with whom we are interacting (i.e., “facial mimicry”) (Hess and Fischer, 2014). In addition to providing feedback on emotions we are experiencing, facial muscle moreover help to recall an emotion. For example, to evoke a positive or negative emotion subjects “re-execute” the motor pattern of the facial expression corresponding to that emotion (Baumeister et al., 2015). Indeed, contraction of facial muscles in response to emotional stimuli is linked with the activation of limbic system and amygdala (Dalgleish, 2004). Interestingly, the reverse is equally true: Hennenlotter et al. (2008) measured a reduced activation of the left amygdala and brain-stem when subjects induced with botulinum toxin attempted to imitate emotional facial expressions.

In Neuromarketing, consumer engagement can be partitioned into a triad of characteristics (behavioral, emotional, and cognitive) that can have positive or negative outcomes on market-related behaviors (Turley and Milliman, 2000; Mattila and Wirtz, 2001). The effectiveness of a marketing stimulus as an exchange of behavioral, cognitive, and emotional factors is directly related to the purchase intention. From a consumer psychology perspective, emotional aspects of decision making have been at the core of our understanding of how consumer choices are made ever since Damasio and Bechara proposed the “somatic marker hypothesis” (Bechara and Damasio, 2005). Since then, multiple studies have demonstrated the relevance of emotional responses to understanding consumer-relevant behaviors from ad memory (Missaglia et al., 2017), brand name (Ramsøy and Skov, 2014), or product preference (Groeppel-Klein, 2005), among others. However, emotions are sometimes negatively related to willingness to pay for a certain product. Incidental emotions are responses that influence the decision we take but are caused by sources outside the decision-making process (for example, an argument with one's partner or a frustrating day at work). They are carried over in the decision-making process without our awareness: for example, an incidental feeling of anger automatically triggers a motive to criticize other individuals in another context (Quigley and Tedeschi, 1996). Indeed, neuroscience studies of financial decisions showed a positive correlation between incidental emotions elicited by a sunny day and the performance of the stock market (Kamstra et al., 2003), or the negative correlation between being eliminated at the football world cup and the stock returns for a specific country (Edmans et al., 2007). To attenuate the effect of incidental emotions in marketing context there are several moderating factors that could be considered. Conversely, integral emotions are related to the decision itself. According to Rozin et al. (1986), integral emotions that are linked to a decision target are difficult to remove, discrediting or benefiting the decision. Integral emotions as part of the “decision-making” process should be related the brain activity in the ventromedial prefrontal cortex (i.e., vmPFC), an important region of the brain for integrating emotion and cognition (Naqvi et al., 2006). One of the roles of neuromarketing is to provide insights able to shape integral emotions to optimize purchasing decisions in the presence of conflicting cognitive information (Loewenstein, 1996; Loewenstein and Lerner, 2003).

Facial expressions are a way to discern the emotional states of customers offering insights into their opinions. Indeed, neuromarketing employs facial electromyography, the Facial Action Coding System (Ekman and Keltner, 1997) through a trained scorer or using a video-capture software that automatically interprets facial landmarks. Marketing research typically relies on self-reports to gather emotional information, forcing individuals to consciously convert their feelings into numerical values with the possibility of introducing “cognitive interferences” (Poels and Dewitte, 2006). Facial expressions have been successfully employed as a non-verbal medium to predict the positive emotional engagement of marketing stimuli when predicting the popularity of YouTube videos (Lewinski, 2015), and in evaluating social media marketing campaigns of two banks over time. However, some authors (Hamelin et al., 2017) used facial expression to relate emotionality of public service announcements with long-term safe driving attitude, concluding that highly emotional advertisements do not impact customer attitude over time compared to low emotional ones.

Recently, doubt has also been raised about facial expressions as a reliable measure of emotional responses. For example, there has been doubts and failed replications of the traditional theory of facial expressions by Ekman (Keltner and Cordaro, 2017), and recent reviews have suggested that facial expressions do not reliably provide sufficient clues to infer emotions from facial movements (Barrett et al., 2019). Indeed, it has been demonstrated that remote facial coding can produce high degrees of false positives and false negatives (Ramsøy, 2015). This implies that the business of using facial expressions as reliable measures of emotional experiences is rather doubtful, and that there is a need for additional research.

The combination of valid and reliable neuroscience with physiology tools to track emotional and cognitive responses can provide solutions for marketers for both off-line and on-line commerce (Ramsøy, 2019). Recently, a new trend of on-line commerce involving virtual reality platforms has emerged, named v-commerce, where web-stores displayed dynamic 3D models of their products (Zhang et al., 2004). However, the future lies in creating immersive VR shops allowing consumers to navigate and interact as in physical ones. Neuromarketing research is trying to simulate these environments to gather information on consumer behavior when immersed in virtual worlds. For example, consumer intentions were analyzed in a virtual grocery when subjects were exposed to tri-dimensionally reconstructed fruits or vegetables (Verhulst et al., 2017). Another recent paper addressed state of the art and future challenges of this emerging sector offering a panoramic view of different immersive techniques applicable in v-commerce (Alcañiz et al., 2019). The adoption of immersive technologies is further encouraged by the rapidly decreasing prices of VR helmets, spurring the potential of v-commerce environments. In virtual settings, future investigations will decode how virtual worlds influence behavioral, emotional, and cognitive factors in their convergence to the decision-making process. Thus, there is an explicit need for solutions that allow low-intrusive, valid, and reliable measures of emotional responses in VR solutions.

### 1.1. Previous Literature on the Relation Between Surface Electromyography From Zygomaticus Major and Emotions

Zygomaticus Major is a muscle of the lower face that has a large cortical representation over the primary motor area (cortical homunculus) (Rinn, 1984). A study based on a large population of women (Larsen et al., 2003) reported that there are mainly two muscular groups of interest for emotion detection: muscles surrounding the eyes (mainly Orbicularis Oculi for frowning) and those of the cheek for smiling (primarily the Zygomaticus Major). The joint activation of these muscles is commonly called Duchenne smile. The authors found a linear relation between these two muscles and the pleasantness of stimul i. Moreover, for affective stimuli, it seems that surface electromyography (i.e., sEMG) changes on the Duchenne smile muscles are present even if they are not visible (Cacioppo et al., 1986; Tassinary and Cacioppo, 1992). This last observation could lead to the conclusion that sEMG is a superior methodology compared to facial expression detection by video capture. In fact, a recent review of facial configurations as universal expression of emotions (Barrett et al., 2019) suggested to re-evaluate facial expression in the daily life context where they are produced together, with how they are perceived in a “sender” and “receiver” relationship.

In the field of Neuromarketing, facial EMG was already proposed in the past as a way to understand consumers' emotional experiences (Bolls et al., 2001) in advertising research. While listening to radio commercials, if the tone of the voice was more positive a greater activation was noticed over the Zygomaticus muscle, while negative tones elicited more frowning activity. In consumer judgment and decision making, “fluency” of information perception can be associated with positive valence and has facial sEMG correlates over the region of the Zygomaticus Major as shown by Winkielman and Cacioppo (2001) with easy-to-process pictures. “Fluency” becomes important when individuals have to decide between products: products with higher fluency are usually preferred (Schwarz, 2004), and the Zygomaticus major could be an indicator of it.

### 1.2. Study Aim : Assessment of Zygomaticus-Based Metrics as Viable for 3D VR Shopping Scenarios

In the present study we tried to evaluate spontaneous muscular activation leading to smiles evoked by screening of musical videos. This study does not consider the social context of smiling, but solely spontaneous smiling in response to a stimulus when subjects are alone. This situation is similar to VR where subjects are isolated from the outer reality. We focused our research on the Zygomaticus major because it is a muscle easily accessible with a low level of intrusion, with a potential application in neuromarketing studies. The corrugator supercilii may provide similar insights into the affective state of a subject (Larsen et al., 2003), yet its suitability is hindered due to the frequent coupling of eye-tracking in neuromarketing studies leading to potential interference in electrode placement. Moreover, the increasing adoption of virtual commerce studies in neuromarketing and corresponding use of VR headsets which occupy the areas surrounding the brows impede the attachment of sEMG electrodes near the eyes. Likewise, the presence of VR headsets limits the usage of video-cameras to automatically detect facial expressions through software able to categorize facial features, as in on-field neuromarketing research with eye-trackers.

For these reasons, we focused on the time course of the zygomaticus major, a paired facial muscle of the cheek area that lifts the angle of the mouth upwards and laterally to allow a person to smile. Usually this area of the face is not occupied by devices used in neuromarketing studies or VR equipment, and requires only a minimal setup of two electrodes for muscular activity detection. Our aims were to provide an alternative method to video-based emotion detection and to find an interpretable connection between the activity of the zygomaticus major and emotional states applying a minimal sEMG montage.

**Figure 1:**
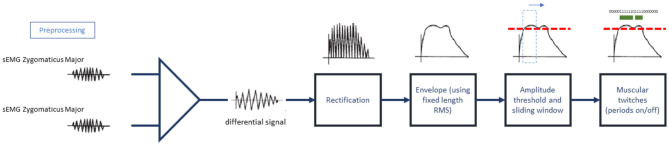
Detection of “on” periods (muscular contractions).

Here, we sum up some advantages of introducing sEMG from the Zygomaticus in Neuromarketing studies for emotional state detection:

- Zygomaticus can be recorded in neuromarketing studies when the eye area is unavailable due to being covered by a VR headset or eye-tracking devices. In this case, the region of the eyes is also hidden to camera-based facial recognition techniques reducing their applicability.
- The Zygomaticus can be recorded from a single cheek with a pair of self-adhesive gelled electrodes (positioned as in Figure 1). This minimal setup could be justified considering that spontaneous smiles representing enjoyment are more symmetrical compared to deliberate smiles (Skinner and Mullen, 1991; Hager and Ekman, 1997). This minimal setup is suitable in case of large-scale studies.
- sEMG, like other neurophysiological techniques, suffers from artifacts, notably power line interference and movement artifacts. Signal processing can reliably isolate spurious activity. Electromagnetic noise is usually suppressed by a notch filter. During head turns, the movement of the cable connecting the electrode to the amplifier could produce a low oscillation on the sEMG signal cancelable by the highpass filter. Both artifacts can be addressed by designing proper filters.
- In women, makeup usually requires stronger algorithms for automatic detection of facial expressions (Moeini et al., 2014) yet sEMG technique overcomes this and only requires the correct application of electrodes with an acceptable contact impedance.

### 1.3. Number of Emotional Dimensions Investigated in This Study

Each participant judged his/her emotional state in response to audio-visual stimuli through questionnaires at the end of video exposure. Participants rated each musical video in terms of arousal, valence, dominance and liking. Arousal and valence are the main dimensions of the circumplex model of affect (Posner et al., 2005): valence is the degree of pleasantness usually related to the frontal cortex, while arousal is associated with subcortical and parietal circuits. Arousal grades stimulus intensity from neutral or boredom responses to high excitement. Dominance is an addition to the classic valence and arousal dichotomy, and attempts to capture psychosomatic aspects of control. It expresses the feeling of control over a given stimulus. Valence, arousal and dominance could be linked in some way to affect, cognition, and behavior in the so-called ABC model or to the concepts of feeling, thinking, and acting (Bakker et al., 2014). Together with the three emotional dimensions, we included Liking scores ranging from 1 to 9 representing how much the participants liked the musical video-clip they watched.

### 1.4. Neural Basis of Emotions

Emotions are conveyed by vision, hearing and touch to the central nervous system to trigger both behaviors and feelings. Feelings of emotions are mental states that follow a behavior caused by external circumstances. Amygdalae are the center of the best know emotion that is fear (Feinstein et al., 2011) while disgust, another human protective emotion, arises from a small portion of the anterior insula (Harrison et al., 2010). Emotions enclosed in the circumplex model of affect seem involving different neral loops. Stimuli evoking arousal showed an activity over the left thalamus, globus pallidus, caudate, parahippocampal gyrus, amygdala, premotor cortex, and cerebellar vermi (Colibazzi et al., 2010), with some differences between genders (Canli et al., 2002) regarding the activation of the amygdala. Valence instead involved midbrain, ventral striatum, and caudate nucleus as portions of a “reward” loop while unpleasant experiences activated supplementary motor, anterior midcingulate, right dorsolateral prefrontal, occipitotemporal, inferior parietal, and cerebellar cortices (Colibazzi et al., 2010).

## 2. Materials and Methods

The dataset used for this analysis is a public-domain benchmark collected by Koelstra et al. (2011). The dataset represents physiological recordings from 32 participants during exposure to 40 musical videos for 60 s each. The videos included different kinds of musical genres. We focused our analysis on the sEMG recordings from the zygomaticus major to highlight muscular patterns in response to the emotional content of the musical videos. Data was recorded at a sampling frequency of 512 Hz with an Actiview software (Biosemi BV, The Netherlands). Electrode placement is shown in Supplementary Figure 1: one electrode was placed one centimeter above the corner of the mouth and the other 1 cm from it following a straight line (blue circles).

### 2.1. Behavioral Data

All participants rated each video on a continuous scale from 1 to 9 in terms of valence (pleasantness or unpleasantness), arousal (boredom or excitement), dominance and liking (Supplementary Figure 2). Authors of the dataset invited participants involved in the study to evaluate their affective reactions on self-assessment manikins (i.e., SAM) for valence, arousal, and dominance. Dominance was intended as a measure of self-control, and participants had to estimate the degree of control musical videos could inspire, ranging from “without control” or helpless to “everything under control” or empowering. For liking scores (i.e., “how much did you like the video?”), researches included icons with thumbs up and thumbs down instead of SAM mannequins.

### 2.2. Pre-processing of Surface Electromyography From the Zygomaticus Major

During the preprocessing stage we initially removed power line noise with a notch filter centered on 50 Hz, and high-passed the sEMG signals at 5 Hz to remove any DC offset. Cut-off frequency of the high-pass filter was selected in accordance with the International Society for Electrophysiology and Kinesiology recommendations for surface EMG (Merletti and Di Torino, 1999). Other publications involving sEMG followed the same standards: for example, in research for muscular prosthetic control (Polygerinos et al., 1996) or in clinical settings for physiatry (Thuresson et al., 2005). After filtering, we calculated the differential mode signal converting the two sEMG traces in a single time series resulting from the difference in voltage between the two recording electrodes.

#### 2.2.1. sEMG Signal Analysis: Muscular Contractions on Whole Window (ZygoNum)

**Figure 2:**
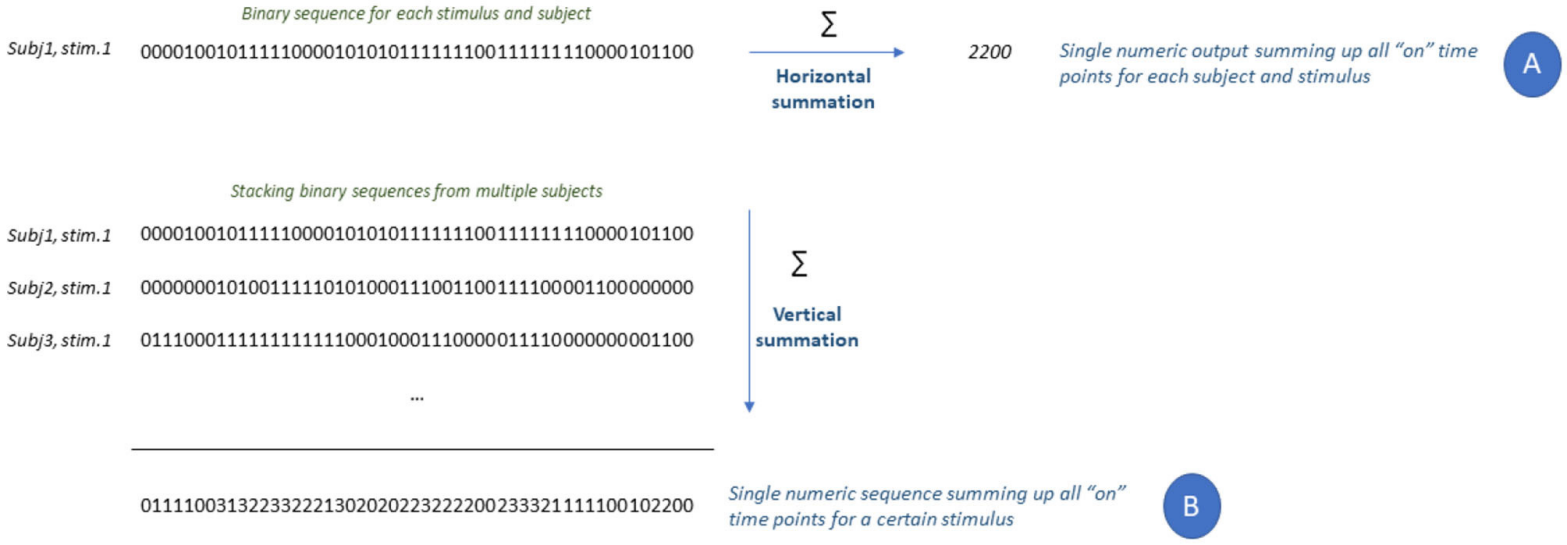
Additive properties of the binary vector.

To highlight time instants during which subjects smiled, the signals were further processed with full wave rectification and envelope calculation. Root-mean-square envelopes were determined with 25 ms sliding window (De Luca, 1997). On the envelopes, we detected the number of times at which zygomaticus muscles turned “on” and “off” using a voltage threshold. Threshold was calculated as μ + J * σ where μ and σ are the mean and standard deviation of the envelope during the 3 s baseline recorded before presentation of each video (period of muscular inactivity). Parameter J = 3 was selected as in Di Fabio (1987). In accordance with (Hodges and Bui, 1996), we counted that window as an “on” period in each 25 ms window when the mean voltage exceeded the threshold, we counted that window as an “on” period (Figure 1). Muscular contractions lasting <125 ms were filtered out to remove micro-expressions. This measure is closer to the upper limit of the original definition of micro-expression given by Ekman and Friesen (1969), Ekman (2009), and Ekman (2003) that quantifies the duration of facial micro-expressions ranging from 1/25 to 1/5 of second. This inclusion limit also removes transient muscular contractions or isolated occurrences of facial tics. The outcome of this procedure is a one-dimensional vector containing a binary sequence with length equal to the original length of the sEMG signal. In the binary sequence, zeros represent time instants where the muscle is “off,” while ones are instants of muscular activation (“on”). The binary vector has two additive properties: binary sequences can be summed up horizontally or vertically as shown in Figure 2. Horizontal summation of all binary values will result in a single score (Figure 2 “A,” called ZygoNum) that could be averaged among subjects to return a general score for a specific stimulus (in our case musical videos). The horizontal summation procedure can be considered as the amount of the time spent by Zygomaticus Major in active state and the time spent smiling by the subject. Instead, vertical summation could show the time instants where subjects smile in the same moment, identifying single events of enjoyment during video screening (Figure 2 “B”). For a practical illustration on the usage of the vertical summation of the binary vector we prepared an example shown in Supplementary Figure 3. In Supplementary Figure 3A, we plotted the valence-arousal plane (Russell, 1980) for each musical video. The green dashed line represents the second order polynomial fit of the data. On the scatter-plot we identified two points with a similar arousal value: one corresponding to a high valence score and one corresponding to a low valence score. We decided for two videos with similar arousal level because induced mood changes fade quickly for arousal but are more consistent over time for valence (Gomez et al., 2009). For the corresponding two musical videos, the sum of the binarised vectors from all subjects was plotted as in Supplementary Figure 3B obtained with vertical summation. Observing Supplementary Figure 3B, it could be noticed that the high valence video had a time interval around 8 s where 5–8 people used to smile for some seconds. In contrast, there are no evident grouped activation in the lower Valence trace. To facilitate the interpretation and visualization of Supplementary Figure 3B we calculated the root-mean-squared upper and lower envelopes of the time series and shaded the area between them. The vertical summation metric will not be used in this work but could be considered for future extension of present findings. Single patterns of muscular contractions could also be analyzed following the procedure exemplified in Figure 2 counting the duration of consecutive “on” instants. We called this measure ZygoLen (Supplementary Figure 4). ZygoLen was mainly added to evaluate, at single subject level, which metric (ZygoNum or ZygoLen) is more effective in detecting emotions and to check whether together they could improve the characterization of affective states (in section 4). Both ZygoNum and ZygoLen provide a single numeric value as output: this characteristic could be useful not only to quantify the sEMG activity with an index, but also because it could be used directly as input feature in combination with other bio-metric indicators for machine learning approaches focusing on emotion detection.

#### 2.2.2. ZygoTrace: Binned Analysis of Zygomaticus Major

**Figure 3:**
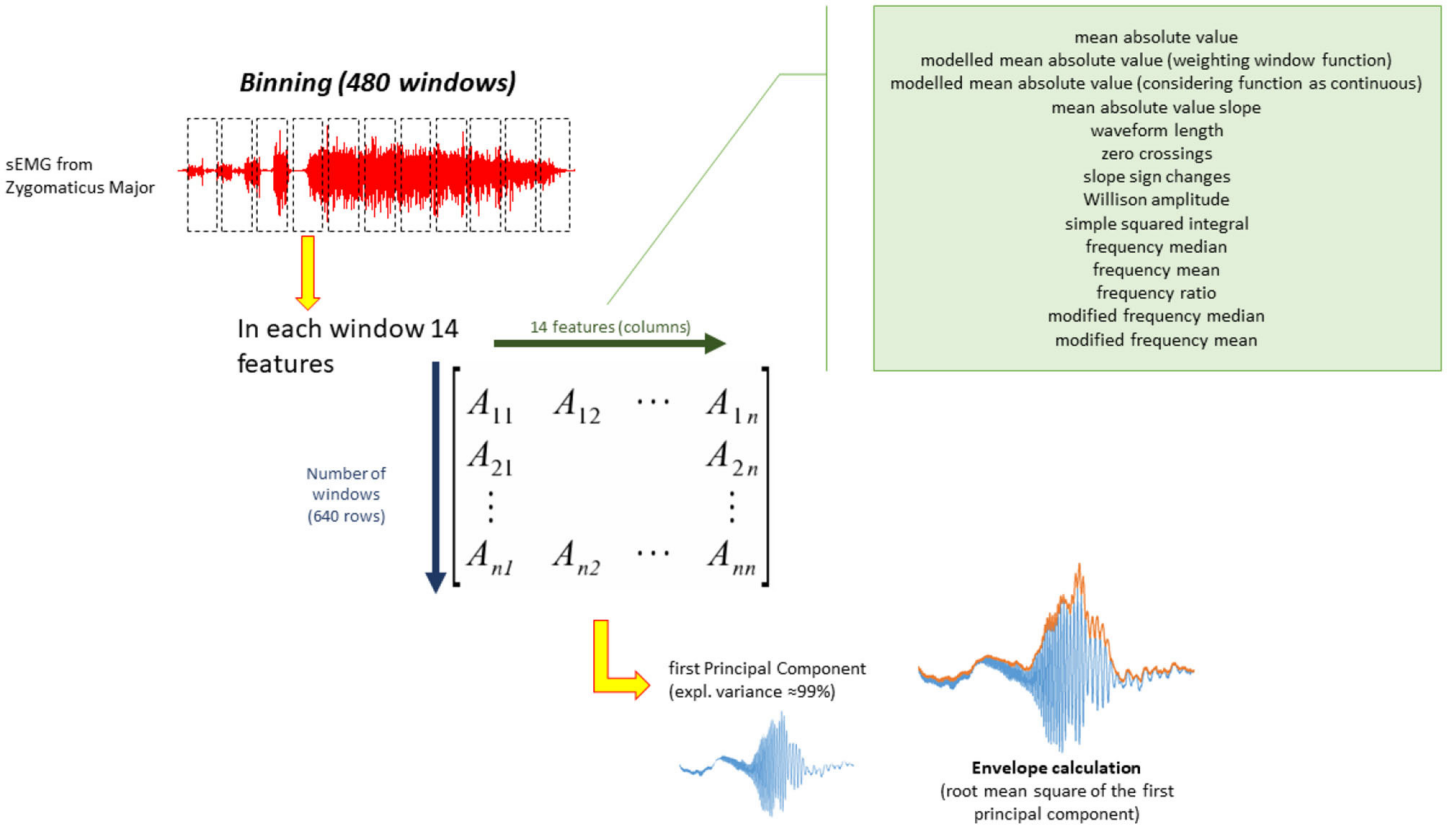
Pre-processing pipeline for binned analysis (ZygoTrace).

We used disjoint segmentation (Oskoei and Hu, 2008) using a sliding window of 64 time points as predefined length (equivalent to 125 ms) to identify a subset of signal segments. In each segment we calculated 14 parameters: mean absolute value of the signal, modeled mean absolute value using a weighting window function (Phinyomark et al., 2012), modeled mean absolute value considering the function as continuous (Phinyomark et al., 2012), mean absolute value slope estimating the difference between neighbor mean absolute values (Phinyomark et al., 2012), waveform length as cumulative length of the waveform over the segment (Englehart et al., 1999), zero crossings as the number of times the waveform changes sign (Phinyomark et al., 2012), slope sign changes, Willison amplitude as the number of times the first derivative exceeds a threshold (Oskoei and Hu, 2008), and squared integral as energy feature (Oskoei and Hu, 2008). In frequency domain, from power spectral density, we gathered information about the frequency median, the frequency mean, frequency ratio (Han et al., 2000), and the modified frequency median and mean (Phinyomark et al., 2012). The time course of the 14 features (640 points each feature) was collected for each subject and each video (32 subjects × 40 musical videos = 1,280 trials in total). To evaluate feature redundancy and retain only relevant information principal component analysis was performed on each trial. It appeared that the first principal component was able to explain nearly 99% of variance in the data. For this reason, the envelope of the first principal component was calculated to simplify the comparisons. Upper envelope was calculated using the root mean square and window length of 16 points. The whole process is depicted in Figure 3.

## 3. Results

**Figure 4:**
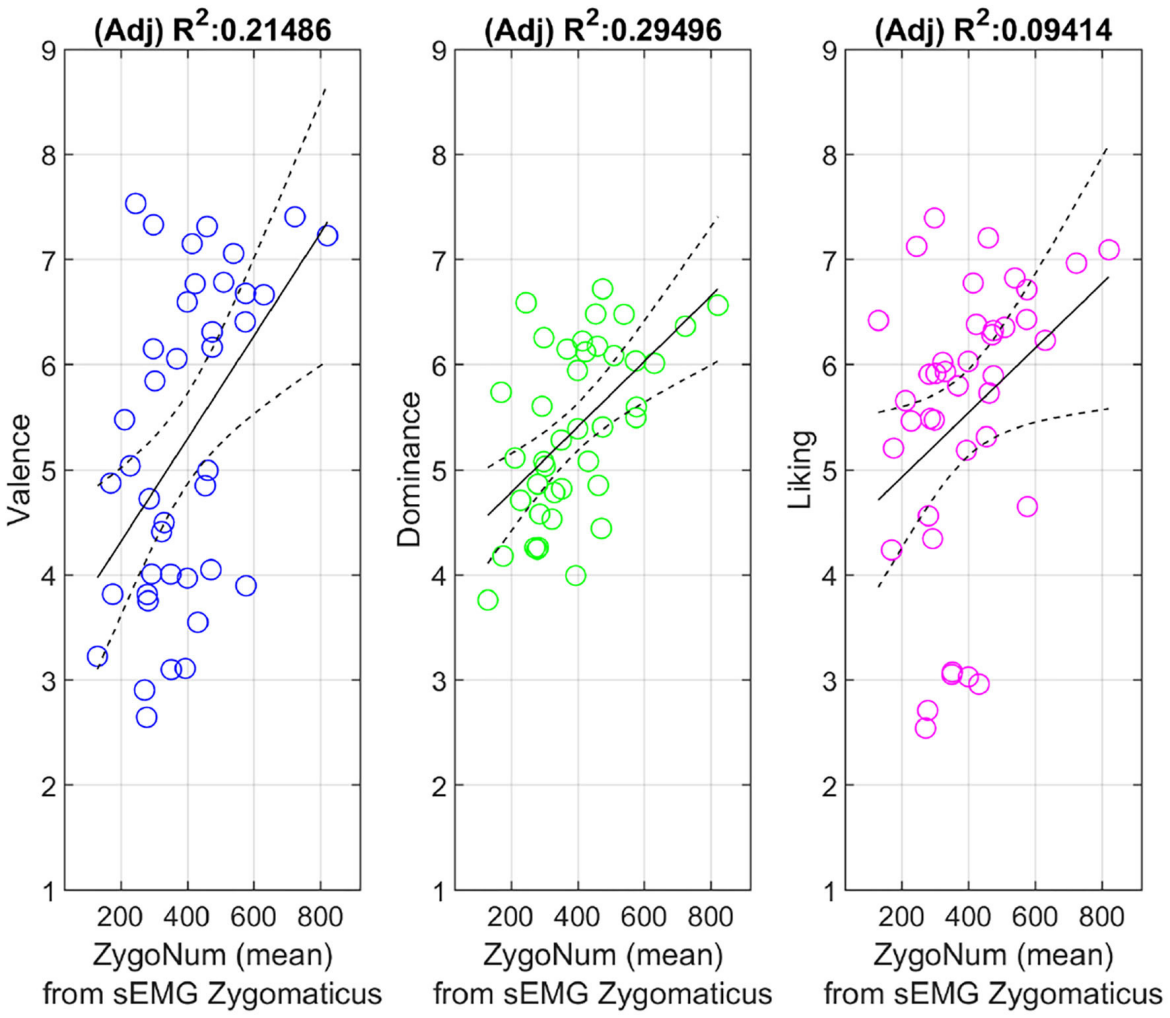
Linear regression models between ZygoNum and Emotional scores for each video (average across subjects). Figures include least square fit and confidence limits.

The sum of all activations (“on” periods) of the Zygomaticus major (ZygoNum) were averaged over subjects and compared to the average scores of Arousal, Valence, Dominance, and Liking as shown in Table 1. The linear dependence is reported with the Pearson correlation coefficient r and the *p*-value testing the hypothesis that there is no relationship between self-reported ratings and number of muscular activations in the zygomaticus major. The number of time instants spent smiling appears to correlate with Valence, Dominance and Liking. Relationships between behavioral data and sEMG activations are portrayed in Figure 4 as linear regressions. Arousal was not included in Figure 4 because it did not reach the threshold of significance and this does not allow us to model a linear relation with the number of muscular contractions. So far, we noticed that ZygoNum has higher correlation with Valence, Dominance and to a lesser extent with Liking. Dominance usually shows lower variance in subjective scores compared to Valence probably because it is more difficult to estimate through surveys: Dominance requires the estimation of an abstract concept like the degree of empowering sensation a musical video could elicit. Valence instead is a more immediate response and its values float more consistently between participants: it could be probably easier for a subject to rate a musical video in terms of Valence using a scale ranging from unpleasant (e.g., sad, stressed) to pleasant (e.g., happy, elated) rather than in terms of Dominance, from “without control” to “under control.” It could be argued that Dominance does not represent a strictly emotional feeling compared to arousal and valence (Russell et al., 1981) and probably it has a cognitive or conative nature.

**Table 1:** Correlation between Self-reported ratings and ZygoNum.

***N* = 40, df = 38**	***r***	***F*-value (*p*-value)**	***R*^**2**^ (adjusted *R*^**2**^)**
Arousal	0.2661	2.9 (0.0970)	0.0708 (0.0463)
Valence	0.4848	11.7 (0.0015)	0.235 (0.215)
Dominance	0.5595	17.3 (0.0002)	0.313 (0.295)
Liking	0.3426	5.05 (0.0305)	0.117 (0.0941)

### 3.1. ZygoTrace for Emotion Detection: Emotional Dimension Detection

**Figure 5:**
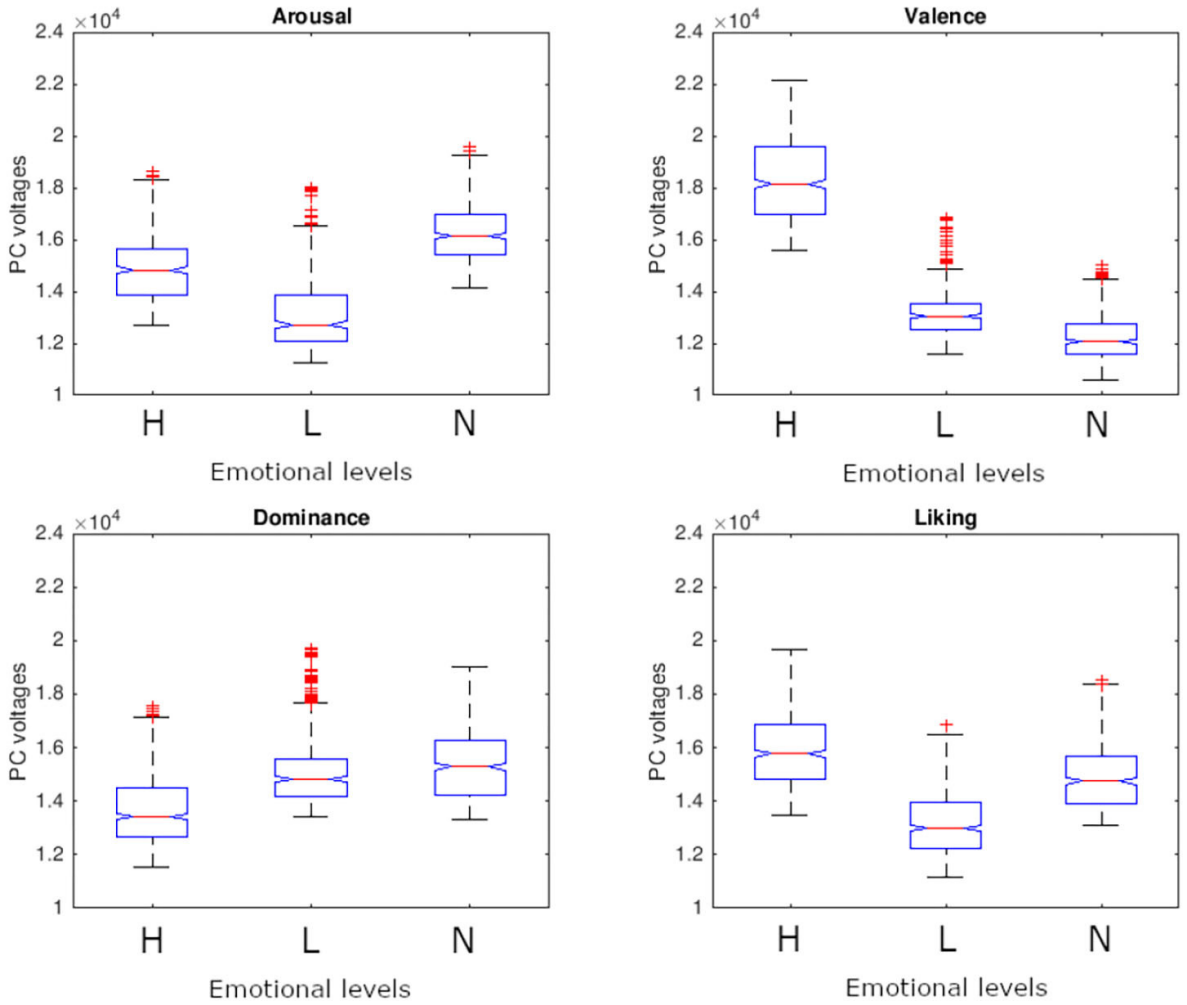
Averaged ZygoTrace grouped by emotional dimensions.

In Table 2, trials are grouped in three levels for Arousal (i.e., A), Valence (i.e., V), Dominance (i.e., D), and Liking (i.e., L): “high” level means an affective ranking above the mean + half standard deviation (i.e., H), “low” level is an affective score below mean minus half standard deviation (i.e., L) and “neutral” level is between “high” and “low” levels (i.e., N). Each group has a similar number of observations to maintain an acceptable level of statistical power.Data distribution of averaged ZygoTrace metric in the three emotional levels (“H,” “L,” “N”) is shown in Figure 5. Analysis of variance to test the effects of the three emotional levels (H, L, and N) on the mean values of ZygoTrace was performed for each emotional dimension (Table 3). The null hypothesis was tested at significance level of α= 0.05 adding “subjects” as random factor to the main factor “emotional dimension” (*n* = 1,280, df = 1,278). It should be mentioned that data comes from a not normally distributed sample (verified with Jarque-Bera test). However, the sample size is large enough to ensure that the non-normality should not impact test's validity (Stevens, 2012). Valence scores were furtherly investigated in a *post-hoc* test with Bonferroni correction. It returned a significant outcome for comparisons between HV vs. NV (*p* = 5.44e-7) and HV vs. LV (*p* = 2.31e-07) while NV vs. LV was not significant. Independent two samples *t*-tests with assumption of inequality of variances were performed for all the groups on the averaged ZygoTrace signal (Table 4). Statistical tests report a significant link between ZygoTrace and Valence. In particular, it seems that ZygoTrace can detect “high valence” stimuli compared to the other two emotional levels.

**Table 2:** Emotional dimensions grouped by score.

	**Count**	**Percent**
**AROUSAL**		
High (HA)	428	33.44
Neutral (NA)	436	34.06
Low (LA)	416	32.50
**VALENCE**		
High (HV)	460	35.94
Neutral (NV)	376	29.38
Low (LV)	444	34.69
**DOMINANCE**		
High (HD)	409	31.95
Neutral (ND)	453	35.39
Low (LD)	418	32.66
**LIKING**		
High (HL)	500	39.06
Neutral (NL)	394	30.78
Low (LL)	386	30.16

**Table 3:** Analysis of variance on ZygoTrace over three emotional levels (H, L, N).

**Emotional dimension**	***F***	***p*-value**
Arousal	1.85	0.1628
Valence	4.61	0.0134 [^*^]
Dominance	<0.0005	Inf
Liking	1.34	0.2677

* *Means p < 0.05 for statistical significance*.

**Table 4:** *t*-test values comparing the averaged ZygoTrace for each group.

	***t*-value**	***p*-value**
**AROUSAL**		
H vs. L	0.89	0.3735
H vs. N	−0.71	0.4719
N vs. L	−1.73	0.0837
**VALENCE**		
H vs. L	2.60	0.0095 [^*^]
H vs. N	3.00	0.0028 [^*^]
N vs. L	0.84	0.3987
**DOMINANCE**		
H vs. L	−0.86	0.3870
H vs. N	−0.84	0.3976
N vs. L	−0.12	0.8973
**LIKING**		
H vs. L	1.47	0.1408
H vs. N	0.54	0.5882
N vs. L	−1.02	0.3073

* *Means p < 0.05 for statistical significance*.

### 3.2. Identified Relation Between Valence and ZygoTrace: Characterization of Their Affinity

Time course of the metric derived from sEMG with the procedure illustrated in Figure 3, is shown in Supplementary Figure 5A. We plotted the time course of the averaged response for each of the three groups. For further analysis, we kept the same categorization of emotional scores in “H,” “N,” and “L” as used before. In the legend of Supplementary Figure 5A we abbreviate Arousal with “A,” Valence with “V,” Dominance with “D” and Liking with “L.” In Supplementary Figure 5B, the nonparametric representation (kernel) of the probability density function of the signals in Supplementary Figure 5A is shown. Observing the distribution of data point for Valence in Figure 3 and Supplementary Figure 5B we can visually appreciate the different distribution of values in the three emotional levels (H, L, and N) as previously highlighted by the statistical tests. Taking advantage of statistical outcomes, we also built a model able to predict the relation between the statistical distribution of ZygoTrace and Valence scores (pleasant vs. unpleasant). Fifteen descriptive statistics were collected from each subject and trial: mean, median, mode, variance, standard deviation, maximal, minimal value, and range between them, inter-quartile range, 5 and 95% percentile, skewness, kurtosis (Stevens, 2012), Hjorth mobility, and complexity (Hjorth, 1970). Mobility parameter is the square root of the variance of the first derivative divided by the variance. Complexity is the mobility of the first derivative of the time series divided by the mobility of the time series. All these parameters were statistically tested one against the other using independent t-tests to assess the significance of each feature in separating the “H” class against the others (we grouped “L” and “N” in one class as direct consequence of significant comparisons shown in Table 4). Neutral and Low Valence were merged in a single class to have a binary classification representing the negative and neutral classes against the “highly pleasant” class. Absolute value two-sample *t*-test with pooled variance estimate are displayed in Table 5 together with their rank. Kurtosis and skewness are the best statistical descriptors because they could be more helpful in categorizing ZygoTrace between “HV” and the other levels of Valence (“LV” and “NV”). Considering a cut-off value of |*t*| = 1.96 (two tailed, df = 1,280, α = 0.05). Inter-quartile range and Mobility could be excluded from further analysis because they do not have enough discriminatory power (they are “redundant” features).

**Table 5:** ZygoTrace features ranking.

**Descriptive statistics**	***t*-value**	**Rank**
Mean	2.88	8
Median	2.81	11
Variance	2.16	13
Maximal value	2.84	9
Minimal value	2.93	4
Range	2.75	12
Inter-quartile range	1.60	14
5% percentile	2.93	7
95% percentile	3.14	3
Skewness	4.25	1
Kurtosis	3.46	2
Mode	2.93	5
Standard deviation	2.84	10
Mobility	1.54	15
Complexity	2.93	6

### 3.3. Predictive Ability of ZygoTrace to Detect “High Valence” Musical Videos Among Others

The predictive ability of ZygoTrace to detect high valence musical videos was tested using a Support vector machine classifier (i.e., SVM) setup for a two classes problem: detecting “High Valence” against “Other levels of Valence” stimuli. The predictive ability was tested on 13 features (same features as in Table 5 except redundant ones) collected on 1280 ZygoTrace time series (32 subjects watching 40 videos). The SVM classifier had its hyperparameters optimized using a Bayesian iterative procedure. We also introduced in the model a penalization parameter to account class imbalance in form of the relative dimension of each class (Supplementary Table 1). The SVM model had a linear kernel function and it was tested in a 10-Fold stratified cross-validation with training size of 1,152 and test size of 128 samples (Supplementary Table 2).

## 4. Discussion

Modern interpretations of facial expressions are reconsidering their connection with emotions. Facial expressions are not just a mirror of the emotional state, but part of the relation between a sender and a receiver (Russell et al., 2003). However, spontaneous facial expression while watching a video streaming on a screen without a human interlocutor may provide a more genuine emotional feedback. Spontaneous facial expressions can be related to a wide range of psychological phenomena but the contraction of the zygomaticus major to raise the angle of the mouth in a smile it has been proved to be a marker of enjoyment in different populations and cultures (Ekman, 1997). Hence, we sought to test the possible relation between the muscular activity of the Zygomaticus Major and four different emotions. Emotions were self-rated using surveys while the muscular activity was recorded using a pair of surface electrodes. We proposed two different ways to interpret the sEMG data from the Zygomaticus called ZygoNum and ZygoTrace. From the binarization of sEMG signals (a sequence of 0 and 1 s) we obtained a vector representing the time instant when the muscle is active (“on” state). We introduced a single score summing up the number of “on” periods of the Zygomaticus (horizontal summation procedure) called ZygoNum. From the binary vector, we also established that it is possible to obtain a time series summing up the sequence of activations from all subjects (vertical summation, time series used only for illustrative purposes in Figure 2 “B”). We also presented a way to extract features from the binned sEMG trace using time and frequency domain parameters. The time series obtained with binned analysis (called ZygoTrace) has characteristics that can identify “high valence” stimuli among others. This feature is important in neuromarketing as highly emotional marketing stimuli are related to stable purchasing intentions over time (Hamelin et al., 2017).

### 4.1. ZygoNum and FACS: Testing “Duration” and “Smoothness” of Smiles

The “Facial Action Coding System” (i.e., FACS) method does not use sEMG, but relies on a trained scorer to measure visible facial behavior or on software to automatically detect facial expressions. Previous reports (Frank et al., 1993) using the FACS found that smiles expressing joy and enjoyment show a smoother contraction of facial muscles compared to non-enjoyment smiles together with a less variable duration of the activation. Authors studied duration of smiles with or without the Duchenne marker (contraction of Orbicularis Oculi) and their findings were confirmed for Duchenne smiles only. Our aim was to test how a minimal sEMG montage can support identification of valence in experimental stimuli and for this reason we could not detect Duchenne smiles. To display Duchenne smiles we should have included electrodes near the eyebrows with potential conflict with VR or other devices involving ocular region. Moreover, in the presence of VR devices, the FACS method to detect facial expressions does not work due to the same physical obstacles. However, we tried to test the outcomes of FACS research with our minimal setup proposing two ways to relate the duration and smoothness of the Zygomaticus with self-reported emotions.

#### 4.1.1. Features Derived From Binarized Signals of the Zygomaticus Major to Evaluate Duration of Muscular Contractions (Single Subject Study)

We introduced a new metric that measures the length of consecutive “on” periods from the Zygomaticus (called ZygoLen). We used this index to test the effectiveness of ZygoNum in discovering emotional states and to examine if a mixed model together with ZygoNum could be more precise for emotional state detection. Moreover, we decided to pinpoint which definition of “duration” could fit better the previous findings on the Duchenne smiles. With these two metrics we can evaluate if the total duration or if the mean duration of the muscular contractions of the Zygomaticus are more effective in identifying the affective outcomes. We also calculated the linear relation at single subject level for each stimuli (1,280 total observations, 1,278 degrees of freedom), as shown in Table 6. The results are weak in terms of generalization because self-reported emotional scores are scattered around ordinal numbers while at averaged level we can consider them as nearly continuous. In addition, emotional responses are not pre-defined but affective reactions are under the effect of various subject-dependent variables. Considering these limitations, we can observe that duration of muscular contractions shows a “trend” with the subjective measures of Valence (for example, Pearson coefficient of correlation is 0.1137 for ZygoNum and 0.0569 for ZygoLen, Table 6). ZygoNum seems to show a score nearly double than ZygoLen in determining stimuli pleasantness. Regarding Liking, metrics have a closer value to each other. We also run a multinominal logistic regression study using duration features of Zygumaticus's sEMG as predictors (ZygoNum and ZygoLen). We used the total sum of muscular “on” periods (horizontal summation, ZygoNum) and their mean duration (ZygoLen) as input features to investigate their relationship with self-reported emotional dimensions (Table 7). To build an ordinal model we had to round self-reported scores (the responses) that were decimals to the nearest integer. In this way the model used the natural ordering of the self-reported emotional scores to describe the relationship between cumulative probabilities of the emotional categories and predictor variables. As link function we used the logit function, assuming that the effects of the predictor variables are the same for all categories on a logarithmic scale. It seems that the original notion of ZygoNum as single number resulting from the total sum (horizontal sum) of “on” time instants could be able to describe a relation with Valence and Liking. Also, in this case, the model identifies a “trend” between smiling time and pleasantness (valence) or liking: more time participants spend smiling and higher could be the Valence or Liking score they will assign for that video. We also report the *p*-values of the interaction terms for the relative probability of being scored a number between 1 to vs. 9 (9 is the highest score the participants could select in the emotional scale and it is our “reference category”). At the end of each stimuli (each musical video), participants rated emotions the video-clip inspired them on a scale from 1 to 9 for all the three emotional dimensions (Arousal, Valence, Dominance) and Liking. In Table 7, we can observe that the total time spent smiling (ZygoNum) has more discriminative power in detecting emotional scores for Valence and at lesser extent for Liking. Using FACS system and Duchenne smiles Frank and Ekman found that enjoyment smiles have less variable duration of activation. We cannot prove this concept using only with the Zygomaticus Major, but we could report a trend between the total number of time instants the Zygomaticus is active (ZygoNum) and the degree of pleasantness felt after watching a musical video (Valence). With less precision, results are extendable to Liking.

**Table 6:** Correlation coefficients at single subject level.

***N* = 1,280, df = 1,278**	**ZygoLen**		**ZygoNum**	
	**r**	***p*-value**	**r**	***p*-value**
Arousal	0.0267	0.91 (0.3403)	0.0337	1.45 (0.2283)
Valence	0.0569	4.15 (0.0418)	0.1137	16.7 (4.54e-05)
Dominance	−0.0076	0.0736 (0.7863)	0.0270	0.934 (0.3340)
Liking	0.0719	6.64 (0.0101)	0.0951	11.7 (0.0007)

**Table 7:** Significant differences (“*” when *p* < 0.05) between emotional scores and the reference category (highest value i.e. 9).

		**1 vs. 9**	**2 vs. 9**	**3 vs. 9**	**4 vs. 9**	**5 vs. 9**	**6 vs. 9**	**7 vs. 9**	**8 vs. 9**
Arousal	ZygoNum	0.1014	0.0746	0.0612	0.0614	0.9279	0.4654	0.5791	0.1385
	ZygoLen	0.4129	0.2915	0.4503	0.7735	0.4661	0.1638	0.8159	0.0941
Valence	ZygoNum	0.2453	0.127	0.0164*	0.0154*	0.0012*	0.0074*	0.0209*	0.0003*
	ZygoLen	0.4726	0.23	0.4311	0.4137	0.6374	0.1064	0.63	0.5129
Dominance	ZygoNum	0.514	0.3581	0.2176	0.0338*	0.1304	0.3985	0.9821	0.3864
	ZygoLen	0.3478	0.4918	0.4613	0.4096	0.8951	0.266	0.1971	0.0389*
Liking	ZygoNum	0.1008	0.1131	0.1218	0.0273*	0.0087*	0.4359	0.0508	0.0004*
	ZygoLen	0.973	0.7428	0.8342	0.9405	0.7049	0.0437*	0.3702	0.5098

#### 4.1.2. Smoothness of Activation Patterns in the Zygomaticus Major

**Figure 6:**
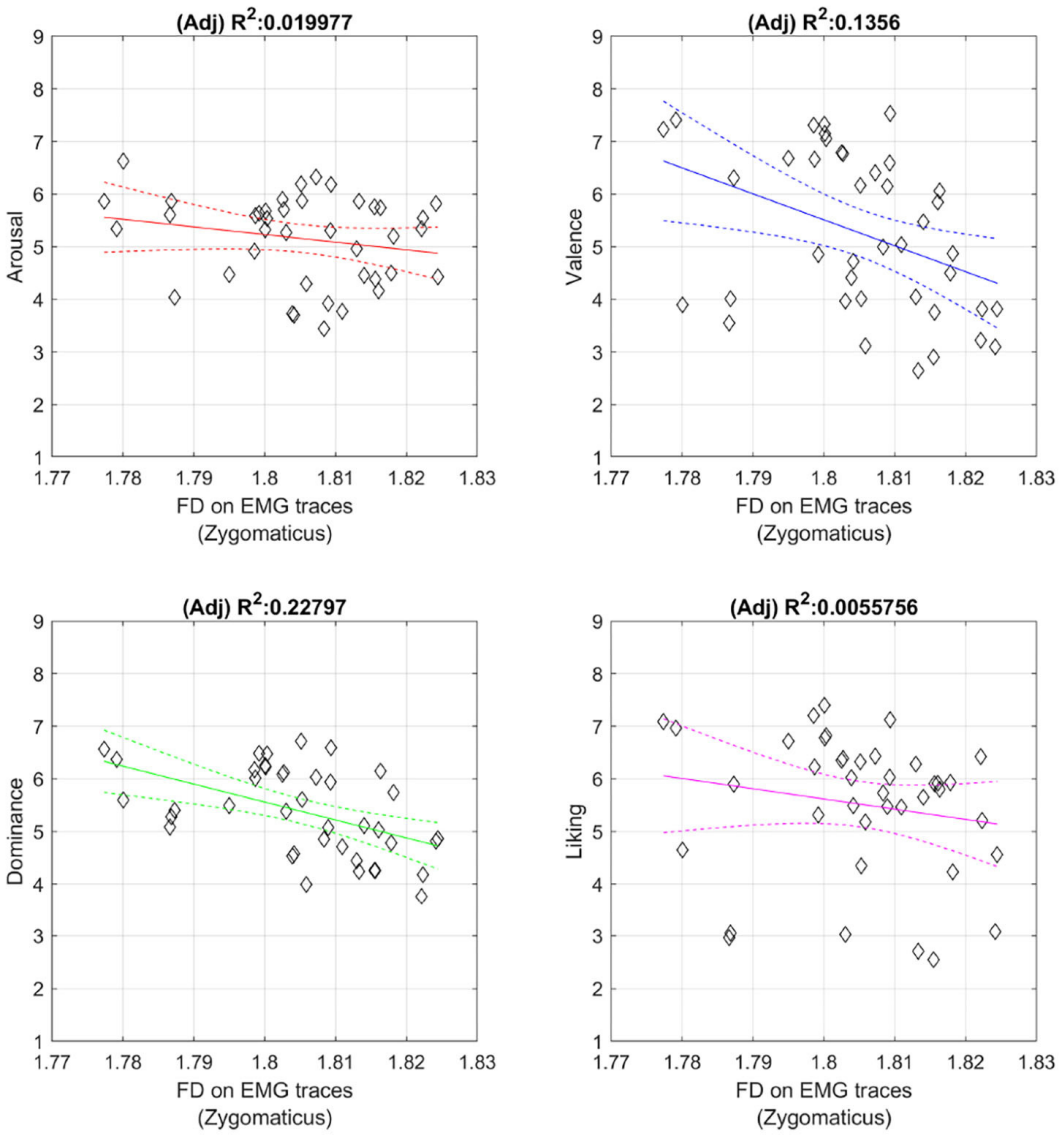
Inverse relation between FD and Emotional dimensions. Figures include least square fit and confidence intervals.

We tested this hypothesis measuring the degree of smoothness of the sEMG activity from the Zygomaticus through Higuchi's Fractal Dimension (i.e., FD). This non-linear method has a long history of application in neurophysiology (Kesić and Spasić, 2016) and we interpreted this fractal measure of irregularity as the degree of smoothness of the sEMG time series. We preferred this method instead of the classic calculation of the number of “onsets” and “offsets” as in Hess and Kleck (1990) because the FACS method does not assume a return to the neutral position (Frank et al., 1993). We calculate Higuchi's fractal dimension on the sEMG envelope previously obtained as in Figure 1 (skipping the last two steps, without proceeding with the sliding window for the “on” period calculations). Each single envelope was down sampled by a factor of 16 to reduce the RAM usage and we calculated Higuchi's fractal dimension using 126 sub-series composed from the original signal. The correlation was measured between the degree of smoothness and the emotional dimension using the coefficient of correlation (Table 8) and fitting a linear regression method (Figure 6). From the data we examined it seems that a certain degree of relation exists between the smoothness of the sEMG from the Zygomaticus and the emotional dimension of each stimuli (only Valence and Dominance have a linear inverse relation with *p* < 0.05). When Valence of the musical video increases the complexity of the sEMG activity from the Zygomaticus decreases, leading to a more smoothed envelope. The models are an approximation and probably the portion of variance they explain is limited but a trend seems to emerge from this investigation: this tendency seems aligned with previous findings using the FACS method.

**Table 8:** Correlation coefficient between self-reported ratings and smoothness of the Zygomaticus major (averaged across subjects).

	***r***	***F*-value (*p*-value)**	***R*^**2**^ (adjusted *R*^**2**^)**
Arousal vs. FD of the Zygomaticus	−0.2124	1.79 (0.188)	0.0451 (0.02)
Valence vs. FD of the Zygomaticus	−0.3972	7.12 (0.0122)	0.158 (0.136)
Dominance vs. FD of the Zygomaticus	−0.4978	12.5 (0.00108)	0.248 (0.228)
Liking vs. FD of the Zygomaticus	−0.1763	1.22 (0.277)	0.0311 (0.00558)

### 4.2. ZygoTrace and Previous Literature: Usage of Zygomaticus for Valence Prediction

ZygoTrace is obtained using a binned analysis: in each window we extracted 14 features in time domain and frequency domain. We processed the data to obtain a single time series using principal component analysis for dimensionality reduction and root-mean-square envelope for smoothing the redundant fluctuations of the signal. From the resulting signal we extracted 13 statistical descriptors and used them as input features of a Support Vector Machines classifier: it resulted that it is possible to detect “High Valence” stimuli with a mean accuracy of 76.63% at cross validation (Supplementary Table 2). We compared our method that uses a single channel sEMG (ZygoTrace) with a recent work on valence classification using a single channel EEG device (Ogino and Mitsukura, 2018). It seems that methods perform similarly, with the single channel EEG valence predictor achieving 72.40% accuracy on binary classification of valence levels (Table 9). Both methods use Support Vector Machines as machine learning approach. Considering the good predictive ability shown by ZygoTrace to detect Valence we could suggest for future research to include sEMG in EEG models for emotion classification, using respectively sEMG to detect Valence and choosing EEG to measure Arousal. In this way the circumplex model of affect that reduces all emotions as a linear combination of valence and arousal (Colibazzi et al., 2010), could be reconstructed with machine learning models using sEMG from the Zygomaticus for Valence and EEG channels for Arousal. Alternatively, sEMG and EEG could be both used as single inputs or as fused or agglomerated input features to enhance Valence prediction. The overall findings that we report on facial EMG seem in accordance with previous literature (Cacioppo et al., 2000) where facial sEMG from Zygomaticus can detect pleasant versus unpleasant stimuli but cannot categorize different emotional states.

**Table 9:** Single channel sEMG vs. EEG for Valence detection.

**Input type**	**Number of classes**	**Targeted emotion**	**Accuracy**
1 channel sEMG (ZygoTrace)	Binary (2 classes)	Valence	76.63%
1 channel EEG (Ogino and Mitsukura, 2018)	Binary (2 classes)	Valence	72.40%

### 4.3. Role of Valence in Neuromarketing

Emotional valence as the concept ranging from attractiveness to averseness has a natural application in marketing and customer choice. In the rationalistic theory of decision making, reasoning leads to the optimal choice optimizing utility for a customer. Under this view, emotions are distortion of the rational thinking due to their irrational nature. However, emotions play an important role in decision making before and after taking a decision (Loewenstein and Lerner, 2003). The emotions felt before taking a decision build the expectations while emotions after the decision is taken have a somatic or outward expression. In our study we evaluated Valence after stimulus presentation, but Valence could have important impact before a decision is taken. For example, in Ma et al. (2010) the Authors asked to the participants of their experiment to rate a picture with negative or neutral valence before viewing beverage brand names and product names of other categories. Outcomes from evoked potentials suggested that “brand extension” (using an established brand name on new products to increase sales) would not be accepted when a negative emotional valence was induced. Similar outcomes are reported in Bastiaansen et al. (2018) where the vision of a video-clip with positive valence showing a touristic destination before judging pictures of the same or other touristic places could have an effectiveness on tourism marketing. Regarding emotional valence, Authors of Bercik et al. (2015) detected valence from EEG signals investigating the influence of lights position and colors on non-packed food displayed in grocery shops. Authors suggest that a correct combination of light position and colors could enhance the perception of a positive valence in the displayed fruit or vegetable. Relation between valence and information fluency was already mentioned in the Introduction: information fluency is important in customer experience because it can lead to increased feeling. Cited papers of neuromarketing literature indicate that valence seems important in product evaluation, brand extension, and information fluency.

### 4.4. Further Notes on sEMG Application for Neuromarketing Studies

- sEMG from the Zygomaticus cannot detect spontaneous smiles covertly: EMG electrodes attached to one's face may induce a less naturalistic behavior. However, a minimal montage of only one muscular channel should minimize this problem. Moreover, we tested this technique on a dataset that evaluates spontaneous smiles of subjects alone in a room while watching musical videos. In this context there are not social biases that could rationally affect our unconscious behavior. During VR, we think that the same pattern could be found because the subject is isolated from the outside world both in terms of visual and audible stimuli.
- Zygomaticus has a large cortical representation (cortical homunculus) but it could have a dominant side (as we have for the hand) and the position of the side of the face where to place the electrode should be evaluated. It should be noted that here we are evaluating only spontaneous smiles outside the social context where facial expressions are usually more complex. It was also demonstrated that spontaneous smiles are less asymmetric (Skinner and Mullen, 1991; Hager and Ekman, 1997).

## 5. Conclusions

In this paper we analyzed the relation between muscular activations of the Zygomaticus major and affective responses elicited by musical video-clips. We proposed two ways to characterize the muscular activity using a minimal setup of one sEMG channel: a duration index extracted from muscular contractions representing the total time spent smiling and another method that produces a time series whose statistical features can detect “high valence” stimuli at 76% of accuracy. From analysis of muscular onsets, we found a connection between the activity of the Zygomaticus and Valence (and at lesser extent with Liking). The relation between the muscular activity of the Zygomaticus Major and the degree of pleasantness was already identified in previous literature and we confirmed previous findings using the proposed metrics. We hypothesize that these metrics could be used in future to detect the degree of pleasantness of stimuli in neuromarketing studies that cannot use computer programs to detect facial expressions. For example, in v-commerce when the area near the eyes is covered by VR headsets, video-capture methods for emotion detection cannot be applicable. For Valence detection while wearing VR headsets, researcher could consider the inclusion of a single channel EMG trace from the Zygomaticus Major.

## Data Availability Statement

Publicly available datasets were analyzed in this study. This data can be found at: https://www.eecs.qmul.ac.uk/mmv/datasets/deap/readme.html.

## Author Contributions

MN processed the data, conceived the metrics and performed the analysis, and drafted the manuscript. TR contributed to the interpretation of the results and revised the manuscript. TR and MN contributed to the final version of the manuscript. All authors contributed to the article and approved the submitted version.

## Conflict of Interest

TR is CEO of Neurons Inc. MN is an early stage researcher hosted at Neurons Inc.
