# Supplementary material for: A Minimal Setup for Spontaneous Smile Quantification Applicable for Valence Detection
Source: Front Psychol. 2020 Dec 17;11:566354. doi: 10.3389/fpsyg.2020.566354 (PMC7773723; doi:10.3389/fpsyg.2020.566354)
Supplement: Supplementary file 1 [file Data_Sheet_1.PDF]

Supplementary Material

1 SUPPLEMENTARY TABLES AND FIGURES

1.1 Tables

Group	Number of instances	Percent
High Valence (H)	460	35.94%
“Other levels of Valence” (N+L)	820	64.06%

Table S1. Frequency table of classes used for SVM classification

Classifier	CV mean accuracy	Stand. Dev,
SVM with linear kernel	76.63%	±0.58%

Table S2. Classification on 13 features derived from ZygoTrace

		ZygoLen		ZygoNum	
	Est. Disp.	Standard Error	t stat (p-values)	Standard Error	t stat (p-values)
Arousal	1.0006	0.0002	-0.55(0.5809)	0.0006	-0.41 (0.6817)
Valence	0.9994	0.0001	-3.62(0.0003)	0.0006	0.15(0.8795)
Dominance	1.007	0.0001	-1.35(0.1759)	0.0006	0.95(0.3387)
Liking	0.9992	0.0001	-2.53(0.0112)	0.0006	-1.01(0.3090)

Table S3. Correlation coefficients at single subject level

1.2 Figures

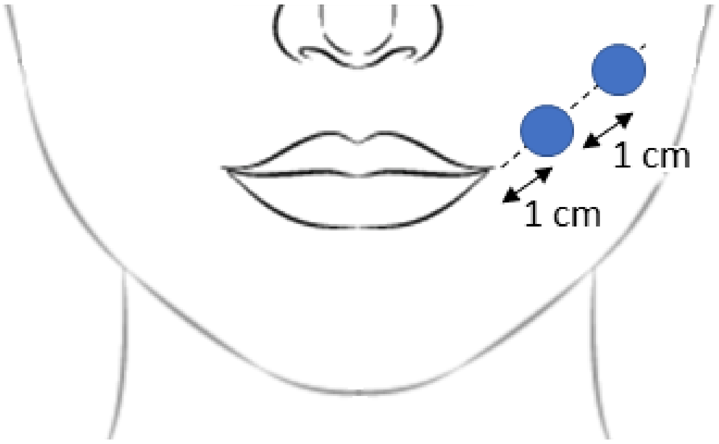

Figure S1. Electrode placement for sEMG recording of the zygomaticus major

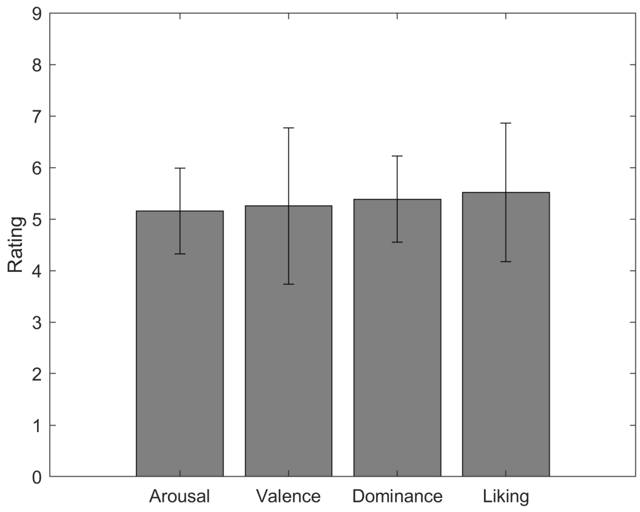

Figure S2. Video ratings (mean and standard deviation as bar plots)

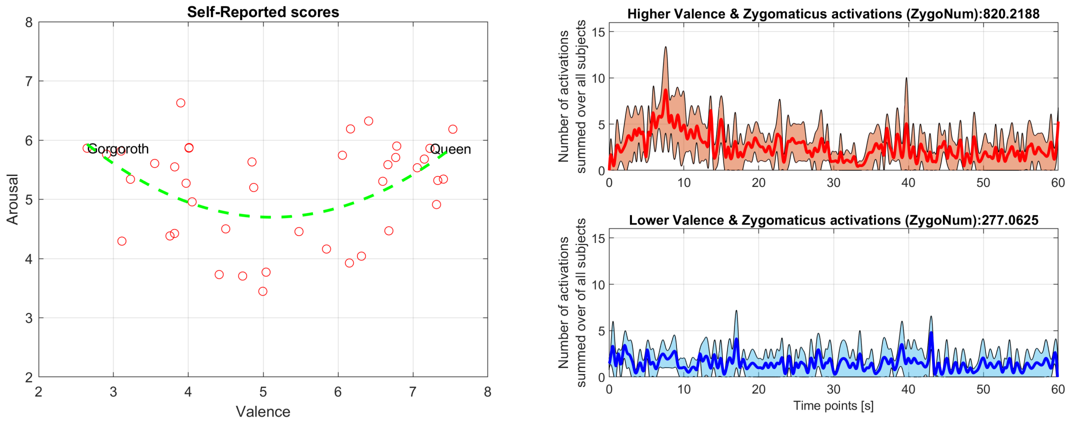

Figure S3. Example of metric obtained by vertical summation from all subjects data, **(A)** Scatterplot of subjective ratings of valence and arousal, **(B)** Time series of ZygoNum (calculated using vertical summation with procedure in Fig. 2 ”B”) from an high Valence video from Queen pop band (upper graph, corresponding to the red line of Fig. S3B) and a low Valence stimuli from Gorgoroth metal group (lower graph, corresponding to the blue line of Fig. S3B)

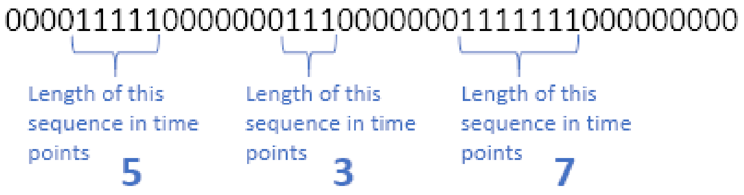

Figure S4. Analysis of the length of single muscular contractions (ZygoLen)

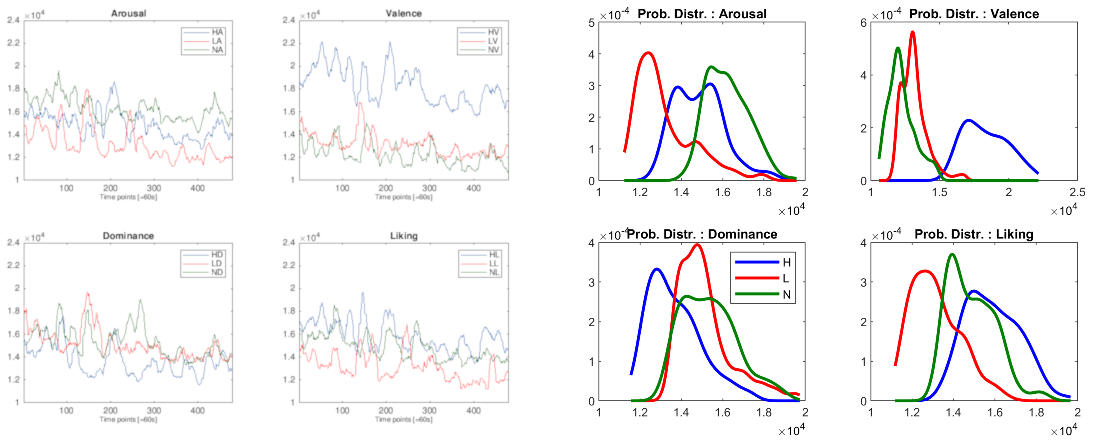

Figure S5. Example of ZygoTrace metric grouped in three emotional levels, **(A)** Averaged time course of ZygoTrace for each emotional state (x axis=time points equivalent to 60s of musical video exposure), **(B)** Probability density function of ZygoTrace for each signal)
